# The seewo interactive whiteboard (IWB) for ESL teaching: How useful it is?

**DOI:** 10.1016/j.heliyon.2023.e20424

**Published:** 2023-09-27

**Authors:** Zhanni Luo, Xiaoyong Tan, Mu He, Xianyou Wu

**Affiliations:** Affiliation School of Foreign Languages and Literatures, Chongqing Normal University, Chongqing, China

**Keywords:** Interactive whiteboard (IWB), Technology acceptance, Perceived usefulness (PU), Teacher perception, Grounded theory

## Abstract

The perceived usefulness (PU) of a technology is critical for its final adoption; however, what makes an interactive whiteboard (IWB) perceived useful remains unclear. This study aims to investigate how pre-service teachers perceive the usefulness of Seewo interactive whiteboards for ESL (English-as-a-second-language) instruction. We recruited 80 pre-service ESL teachers, divided them into 16 groups, provided five weeks of training, and conducted focus group interviews to gather qualitative feedback. The data were analyzed using grounded theory techniques with the aid of the software Nvivo. Three research questions were addressed: how pre-service ESL teachers perceive the usefulness of Seewo IWB in terms of its functions that enhance the learning process (PU-process), how they perceive the usefulness of Seewo IWB in terms of its functions that improve learning outcomes (PU-outcome), and how they perceive the non-usefulness of Seewo IWB. The findings shed light on the determinants of the perceived usefulness (PU) concept, which is crucial for fostering a higher intention to accept technology.

## 1 Introduction

### 1.1 Research background

Due to their potential to engage learners and enhance learning achievements [1], educational technologies have garnered considerable investments from governments and educational institutions [2,3]. Among the diverse array of educational technologies, the interactive whiteboard is a popular choice [1,4].

An interactive whiteboard, abbreviated as IWB, is defined as “a large, touch-sensitive board which is connected to a digital projector and a computer” [5]. A key feature of an IWB is its ability to “control the computer at the touch of the screen” [6]. IWBs offer functions that facilitate teaching and learning, such as accessing the internet for additional educational resources, sending screenshots to learners, and magnifying images for improved readability [2,5].

Technology acceptance studies are important: no matter how promising one technology is, its potential cannot be realized if it is not accepted and implemented [7,8]. Among various frameworks, the Technology Acceptance Model (TAM) proposed by Davis [9] is a popular one [10]. According to the TAM model, users' technology acceptance behavior is affected by attitude, which is determined by the technology's perceived usefulness (PU) and perceived ease of use (PEoU).

### 1.2 Research gaps

There is a scarcity of research exploring the nuances of the perceived usefulness concept, specifically the distinct benefits of an innovation. Davis [9] posits that if a technology is believed useful, it is more likely to be accepted by users. However, the factors that contribute to users believing a technology is useful and the technology's benefits remain unclear. This gap in understanding extends to the reasons behind users' resistance to adopting a specific technology, how to bolster their motivation, and how to minimize barriers [11].

Furthermore, the concept of “perceived usefulness” itself warrants an update. Bourgonjon, Valcke, Soetaert and Schellens [12] argue that it is overly narrow to focus solely on academic outcomes when evaluating the perceived usefulness of educational technologies, as “education means more than mere outcome” [12]. Accordingly, Bourgonjon, Valcke, Soetaert and Schellens [12] divide the PU concept into two sub-concepts: the perceived usefulness of an innovation in augmenting expected outcomes (primarily academic achievement) and the perceived usefulness of an innovation in enhancing the learning process (e.g., stimulating learning interests, enhancing learning engagement). In the present study, the authors have reframed these two themes as PU-outcome and PU-process (see Table 1).

**Table 1:** The PU-outcome and PU-process concepts.

#	Concept	Definition
1	PU-process	The perceived usefulness of a technology lies in its potential to enhance the learning process, such as improving learning interests, enhancing engagement, and reducing anxiety.
2	PU-outcome	The perceived usefulness of a technology lies in its potential to enhance expected concrete outcomes, such as improved academic achievement and behavioral changes.

At the same time, the concept of interactive whiteboards continues to evolve, yet academic research struggles to keep pace [13]. Nowadays, companies in this field are not only investing in hardware enhancements, such as touch screen sensitivity and multi-touch recognition accuracy but are also persistently developing supplementary software systems, with the goal of establishing a comprehensive interactive ecosystem. For instance, the Seewo company offers 11 hardware tool products and 22 interactive courseware production tools. These 22 tools include options like “SeewoCare”, which, similar to the popular gamified software ClassDojo, provides personalized feedback for students; “Seewo Pinco”, a classroom response system comparable to the gamified software Kahoot! and “Seewo EasiNote”, a PowerPoint-like software enabling teachers to design educational interactive games. It's difficult to name these software systems as interactive “whiteboards”; instead, they represent a collection of software that enhances the interactivity of interactive whiteboards. Regrettably, this aspect has not received due attention in academic research.

Furthermore, there is a scarcity of qualitative research that delves deeper into the Technology Acceptance Model (TAM). According to, the majority of research studies on the Technology Acceptance Model have relied on quantitative methods, leaving a gap in our understanding of the subject. Quantitative research in the technology-acceptance field tends to be retrospective rather than predictive [14], which constrains the development of technology-acceptance research.

### 1.3 Objectives and research significance

This qualitative study aims to explore pre-service ESL teachers' perspectives on the perceived usefulness of interactive whiteboards (IWBs) in ESL teaching. The authors collected qualitative data from 80 pre-service ESL teachers and categorized the data into three main aspects: perceived usefulness in enhancing the learning process (PU-process), perceived usefulness in enhancing the learning outcomes (PU-outcome), and perceived non-usefulness.

This study is significant because it is one of few that investigate the determinants of the PU concept. Although PU is a main construct of technology acceptance [9,15], few studies have specified what makes users perceive a technology as useful. Specifying the factors helps promote IWB acceptance, as the education leaders can enhance the motives while eliminating the barriers [16].

In addition, measuring the perceived usefulness of a technology is crucial, and thus, scholars are endeavoring to establish and validate a PU scale [11]. The creation of the initial item-pool is particularly critical [17]. The results of this paper offer valuable firsthand data for developing these scales, enhancing their comprehensiveness, integration, representativeness, reliability, and validity.

Moreover, in previous research, the usefulness of electronic interactive whiteboards has been a highly debated topic. This study emphasizes the importance of differentiating perceived usefulness into two separate concepts: PU-process and PU-outcome. This differentiation can significantly clarify the controversy surrounding the previous research findings.

This study is of great significance because it broadens the definition of “interactive whiteboard” from a hardware-centric perspective to one that encompasses supplementary software. Prior research has primarily focused on touch-sensitive electronic boards, whereas we employed two software programs provided by Seewo company (Seewo Pinco and Seewo EasiNote) in our exploration of ESL teachers' perceptions of interactive whiteboard systems that incorporate both hardware and software.

Another research significance of the current study is the emphasis on negative case analysis. Negative case analysis is a methodology to “analyze cases in which an outcome that had been predicted by theory did not occur” [18], which improves the trustworthiness of grounded theory methodology research [19]. As a result, the *perceived non-usefulness* of Seewo IWBs was also explored.

These findings provide valuable insights for leveraging or enhancing educational technologies in a more effective manner, thereby enabling these tools to better serve teaching and learning. Instead of blindly adopting or rejecting these technologies, these results can inform a more deliberate and informed approach to utilizing them in educational settings.

### 1.4 Research questions (RQs)

Because there are considerable differences in subject matter in education and teaching, the research scenario for this study is using the Seewo Interactive Whiteboard (IWB) to teach English as a second language (ESL). This study seeks to answer the following three research questions.

RQ 1 What connotations do pre-service ESL teachers associate with the perceived usefulness of Seewo IWB in improving the learning process (PU-process)?

RQ 2 What connotations do pre-service ESL teachers associate with the perceived usefulness of Seewo IWB in enhancing learning outcomes (PU-outcome)?

RQ 3 What connotations do pre-service ESL teachers associate with the perceived non-usefulness of Seewo IWB?

## 2 Literature review

### 2.1 Interactive whiteboard and technology acceptance

Interactive whiteboard technology, often abbreviated as IWB technology, is characterized as a large, touch-sensitive board connected to a computer and a projector. This setup allows for the display of text, images, animations, and videos [5,6,[20], [21], [22]]. Schmid [23] explains that fully-functioning interactive whiteboards typically consist of four components: a computer, a projector, suitable software, and a display panel, which is either a large free-standing or wall-mounted screen. Being described as an electronic whiteboard connecting with a computer [6], an IWB contains the basic functions of a computer, such as connecting to the Internet, showing video clips, and demonstrating a piece of software [5].

With the continuous development of the industry, the IWB concept expanded as well. At the first beginning, IWB only allows one user to use fingers to leave marks or write notes on the surface. At that stage, research focuses include plug-and-play installation of the video conferencing system, one-click connectivity, lag-free interactive display and hardware infrastructure refinement (like 4K camera, 16-element microphone, top-notch speakers, etc.). Then, companies devoted to develop multi-touch screen that allows more than one people to interact with the board at the same time. For example, the product SamSung Flip enables four users to interact with the electronic board synchronously.

After that, the IWB industry presented a new tendency: developing IWB software to better support the hardware. Even though an IWB is connected to a computer so it per se enables users to get access to any software that are available on the computer [21], the software may not able to provide true interactive experience. Companies attempted to fill the gap by designing software specifically for interactive whiteboards, which normally contains interactive texts and activities and “is illustrated with colourful graphics and sound effects” that engage users [21]. Taking the well-known electronic interactive whiteboard company Seewo as an example, they offer 11 hardware tool products and 22 interactive courseware production tools. However, these attempts are being driven by the industry, while academia has not kept up.

In academia, technology acceptance studies are important, because they help us understand why people adopt or reject new technologies [10]. With this understanding, we can identify factors that may influence technology adoption and develop strategies to promote the adoption of beneficial technologies [24]. Technology acceptance studies can also help technology developers create products that meet the needs and preferences of their users. Ultimately, technology acceptance studies can contribute to the successful implementation of new technologies in various settings.

### 2.2 Benefits of using IWB for classroom-based teaching

The existing body of research has demonstrated the multitude of benefits associated with the use of interactive whiteboards (IWBs) in student learning, including improved engagement, learning performance, and learning efficiency [22,25]. Further assert that while traditional blackboards tend to place the teacher at the center of the teaching process, IWBs have the potential to facilitate more student-centered and productive learning experiences, ultimately leading to increased enjoyment and motivation. As a versatile tool for multimodal teaching, IWBs can display a variety of media formats, such as audio, video, and animation, thereby enriching the learning experience by integrating multiple sensory channels. For example, when teaching English as a second language, educators can utilize IWBs to showcase multimedia resources, including English-language short films, images, and vocabulary animations, to foster well-rounded development of students' listening, speaking, reading, and writing skills [1,5].

IWBs provide scaffoldings for students with diverse learning styles, as evidenced by studies conducted by Abuhmaid [26] and Schmid [27]. Research indicates that students exhibit varying characteristics and preferences when processing information [28,29]. IWBs cater to these differences by allowing verbal learners to see text, visual learners to see images, and auditory learners to hear sound. Furthermore, Türel and Johnson [30] highlight the benefits of touchscreens, which enable tactile learners to touch and feel the materials as they learn. In a similar vein, research suggests that kinaesthetic learners benefit from physical movement during learning.

In addition, IWBs offer a time-saving advantage since they are essentially computers that can connect to the internet, allowing users to seamlessly access multimedia resources [27,30,31]. With a plethora of multimedia resources available, teachers can use existing materials on the board, thereby reducing the need for extensive material-preparation and lecturing [32].

The use of IWBs also encourages both teachers and students to integrate educational technology into their classroom activities, promoting modernization of the education system [31]. Furthermore, the adoption of new technology allows schools to position themselves as pioneers in the field [26], thereby enhancing their reputation in the education community.

### 2.3 Challenges or problems of using IWB for classroom-based teaching

Some scholars have expressed doubts regarding the effectiveness of IWBs in enhancing student engagement. While students may initially be attracted to the visual stimuli provided by IWBs, their interest may wane once they become accustomed to it and crave new forms of stimuli. This limited range of stimuli, consisting primarily of sound and special effects, can lead to decreased interest and boredom among students [33]. Moreover, the use of external stimuli to improve engagement can result in extrinsic motivation, which research has shown can undermine intrinsic motivation. Türel and Johnson [30] argue that IWBs are primarily a presentation tool in a teacher-led setting, with teacher-student and student-student interactions serving as mere embellishments. This may result in a deterioration of students' learning motivation and engagement over time.

Scholars have expressed concerns about the level of interactivity that interactive whiteboards (IWBs) offer. Research has shown that the interactivity of IWBs is often overestimated, as some teachers only use them to display content, rather than for interactive teaching [20,30]. Schmid [33] suggests that the use of IWBs may lead to a return to the transmission model of teaching from the last century, as teachers plan each step and present pre-prepared knowledge through the use of drill and practice exercises, rather than creating an interactive and generative teaching environment aimed at fostering a free and highly flexible classroom. In other words, the use of IWBs primarily represents a change in teaching media, rather than in social form. Olivares and Castillo [34] add that the lack of true interactivity in IWB classrooms is often due to reduced planning time and the various roles that teachers must fulfill in schools, which hinder them from becoming more familiar with technological tools and lead them to follow the routine and structure of a traditional class. Educational reform requires additional effort, and there is currently no clear incentive to encourage teachers to engage in educational reform.

Olivares and Castillo [34] highlight the playful aspect of interactive whiteboards, stating that new teachers, particularly those who lack advanced teaching and technology knowledge, view IWBs as a gaming tool, and their primary reason for using them is for the entertainment and enjoyment they offer. In contrast to teachers who use IWBs as traditional blackboards, the teachers in their study utilized more advanced IWB features, but ultimately, only used them on a surface level without genuinely creating an interactive learning experience.

Teacher education regarding IWBs presents significant challenges, as many teachers lack knowledge and training in this area. Firstly, teachers often receive limited IWB training from suppliers that only covers basic IWB functions [30]. Secondly, teachers may struggle to integrate IWBs with teaching content. In the study of Abuhmaid [26], teachers reported that mentors did not have the knowledge to assist them in integrating IWBs into their teaching subjects. The integration of new technologies with pedagogical content has always been a challenge [[35], [36], [37]]. Teachers require customized training that is closely related to their specific teaching context, and this training should be immediately applicable [16,26]. However, it is difficult for suppliers to provide such highly customized services.

Studies have also shown that teachers lack confidence in using IWBs in the classroom. The unified theory of acceptance and use of technology (UTAUT) theory suggests that individual acceptance of new technology is influenced by personal innovativeness, which is largely related to age [38]. As a result, younger student teachers may be more willing to adopt new educational technologies [39], but they often rely on their intuition to explore and conduct teaching practices on their own, with few opportunities to collaborate and share good practices [16,26]. This lack of collaboration and sharing of teaching experiences contributes to teachers' lack of confidence and trust in using IWBs in the classroom.

The cost is the biggest obstacle to the adoption of interactive whiteboards (IWBs) [31]. Due to cost issues, not every school can afford the hardware system required for IWBs, and software purchases and renewals may also be prohibitive. In addition, technical support may be gradually phased out due to budget constraints, further raising the barriers to adopting IWBs. The cost issue is particularly pronounced when teachers are unable to fully leverage the advantages of IWBs. Therefore, cost is a major challenge in the adoption of IWBs.

In addition to cost, research has indicated that using electronic whiteboards requires more preparation time, which can also hinder the promotion of IWBs [1,3]. The additional preparation time can be a challenge for teachers who already have busy schedules and limited time for lesson planning. Therefore, addressing the preparation time issue is another important consideration for promoting the adoption of IWBs.

In summary, this study provides an overview of the benefits, challenges, and problems associated with using interactive whiteboards (IWBs) for classroom-based teaching. The literature reveals that IWBs offer numerous benefits, including enhancing student engagement, performance, and learning efficiency, providing scaffolding for students with diverse learning styles, saving time, promoting educational modernization, and improving the school's reputation. However, several challenges and problems exist. Scholars have raised concerns regarding the effectiveness of IWBs in improving student engagement and providing an interactive learning experience. Teachers often lack familiarity with the various functions of IWBs and face difficulties integrating them with pedagogical content. Moreover, some teachers lack confidence in using IWBs, and the high installation and maintenance costs of IWBs serve as significant barriers to their adoption. The cost issue is particularly acute when teachers cannot fully utilize all the functions of IWBs.

## 3 Methodology

### 3.1 Research objectives

This study is to investigate the Seewo interactive whiteboard hardware system and its accompanying software packages. The hardware system of Seewo IWB includes several electronic touch-sensitive boards (infrared interactive whiteboards), an interactive flat panel, a recording and broadcasting system, multimedia speakers, an omni-directional mic, a document camera, a tracking camera, etc. These are installed in a classroom with a podium, blackboard, and student seats, providing the most basic conditions for face-to-face teaching. The supplementary software mainly consists of two programs: Seewo Pinco and Seewo EasiNote.

Seewo Pinco is designed to simulate traditional classroom teaching using a blackboard. It provides basic functions such as writing and erasing on the board, as well as advanced technological features like image insertion, cloning, quick board clean-up, chalk color changes, screen capture, and screen sharing. All of these operations can be executed by touching the electronic screen, thus enhancing the interaction between humans and technology.

Moreover, Seewo has customized an “interaction” toolkit for Seewo Pinco, which includes a voting system. With the voting system, teachers can display multiple-choice questions on the board, and students can select their answers using their personal devices. The answer distribution is then presented on the board. Students can see whether they chose the correct answer and how many of their peers selected each option. Teachers can also assess how well students have mastered the content and provide customized feedback immediately. This voting system is known as a student response system (SRS), also referred to as clickers or classroom response systems (CRS). By allowing students to interact with the teaching content in real-time through their personal devices, the voting system ultimately strengthens teacher-student interactions.

**Fig. 1:**
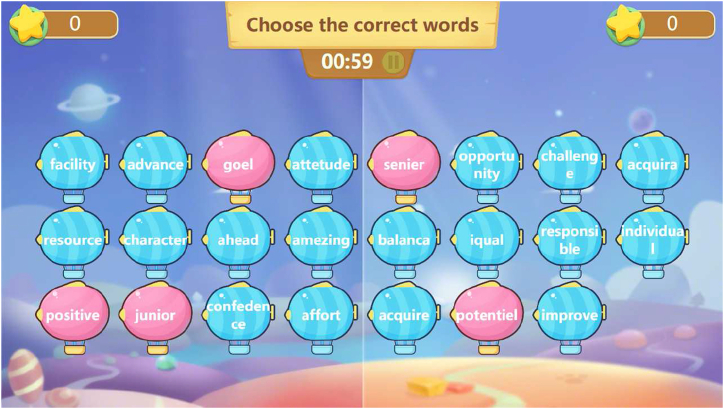

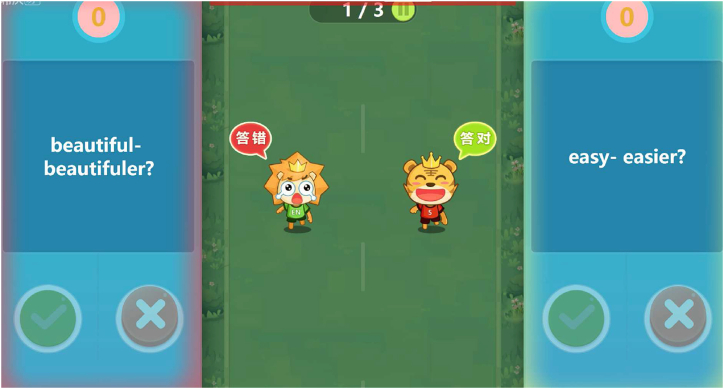
The screenshots of two game-like activities adapted from the templates provided by Seewo EasiNote.

Seewo EasiNote simulates PowerPoint-based multimedia teaching in the classroom, so it looks like the software Microsoft PowerPoint. It includes additional features such as cloud storage, material sharing, mind map drawing, and English dictation. One of the most striking features of Seewo EasiNote is the game-like activity toolkit, which allows users to create interactive games using provided templates (refer to Fig. 1). For example, as shown in the left image in Fig. 1, teachers can prepare a set of English words, including those with incorrect spellings (represented by red icons). During the game-like interactive activity, the English words will appear one by one, and users are expected to identify the wrongly-spelled words by touching the corresponding icons using their fingers. The screen is divided into two halves, allowing two students to interact with the electronic board simultaneously. Points are awarded or deducted based on accuracy.

Seewo offers additional software programs, including ExceedShare, a multi-screen interactive app for wireless content sharing via QR code. However, its limited functions led to its exclusion. Another program, SeewoCare, similar to ClassDojo, enables teachers to create customizable evaluation systems and provide real-time feedback for students. Despite its popularity, SeewoCare focuses on individualized feedback rather than human-technology interactions and the electronic whiteboard's hardware system, so it was not selected.

In summary, we selected Seewo Pinco and Seewo EasiNote as they are the most widely used, multi-functional software programs specifically designed to enhance the Seewo electronic IWB experience.

### 3.2 Participants and research procedure

This study aims to investigate pre-service ESL teachers’ perception of the usefulness of Seewo IWBs. Participants were 107 pre-service ESL teachers that take the course *Educational Technologies and ESL Teaching*. At the beginning of the course, we informed them that there was a research project that they could voluntarily participate in, which focused on ESL teaching using interactive whiteboards.

Before gathering participants' perspectives, we ensured a comprehensive understanding of IWB technology: we offered two introductory classes on Seewo IWB technology, a pair of hands-on workshops for learners to engage with the device, and a session dedicated to facilitating in-depth discussions. Each class lasted 45 min, with the entire training spanning over a five-week period.

Following the five-week training, we posted an advertisement in the learning community that included program information, interview procedures, and an email address for contact. Students volunteered to participate in the study by sending an email request. Out of the 107 pre-service students, 97 responded to the advertisement, and 80 ultimately took part in the subsequent focus group interviews, yielding a participation rate of 75%.

Of the 80 pre-service ESL teachers, the majority were female (76 out of 80, or 95%). Almost all participants were between the ages of 21 and 25. Approximately one-quarter (n = 21, 26.25%) reported having substantial teaching experience, such as more than a year, while a notable 73.75% (n = 59) indicated they possessed limited experience in ESL teaching.

Ethical considerations were carefully addressed throughout the data collection process. Participants were explicitly informed of their right to withdraw from the interview and retract their data up to six months after data collection. Prior to data collection, we provided a comprehensive explanation of the project, presented a clear information sheet, and obtained signed consent forms from all participants. The entire process was anonymous and voluntary. Approval was obtained from the Academic Research Ethics Committee (AREC) of the School of Foreign Languages and Literatures in Chongqing Normal University (approval number: AREC2022SFLL102907).

### 3.3 Data collection and research instrument

During data collection, the 80 pre-service ESL teachers were randomly divided into 16 groups. We conducted focus group interviews for each group in sequence. Each group member received a handout, which included an introduction to the topic, the purpose of the discussion, the rules for focus group interviews, and the specific interview questions. Each group was equipped with a trained moderator to facilitate the discussion.

During the focus group interview, the moderator introduces the research program, obtains participants’ permission, asks questions, and encourages participation from all members. The moderator needs to keep the discussion focused, but allows for flexibility and spontaneity. Each group discussed for 20–35 min.

In line with recommendations of Seidman [40], we refrained from posing the precise question, “What are the determinants of perceived usefulness of the interactive whiteboard?” to avoid leading or implying responses. Instead, we inquired about the teachers' experiences through three questions: 1) what are the pros and cons of employing IWBs in ESL teaching? 2) what are the driving factors and obstacles in utilizing IWBs for ESL instruction? And 3) which features of IWBs have left a positive or negative impression on you during ESL teaching? Given our use of grounded theory techniques, where research findings have minimal correlation to the specific questions asked of participants, these indirect inquiries are appropriate.

The data collection was conducted in Chinese language. The interview responses were recorded, transcribed into text, and then translated into English. Before translating, we conducted data purification, which involved removing verbal fillers, repetitive sentences, and irrelevant responses. During the translation process, we also verified the grammar. Back-translation was used to ensure the accuracy of the translation.

### 3.4 Data analysis and research reliability

The focus group discussions generated qualitative responses, which were analyzed in the software Nvivo using grounded theory techniques. The grounded theory helps establish a unified theoretical explanation for participants’ individual stories [41,42], which is suitable when little is known about what is being studied [43]. This qualitative study aims to comprehensively analyze the sub-dimensions of the PU-process and PU-outcome concepts. However, we have not found any research that dissects these two terms, making it difficult for us to identify a suitable model that explains the factors influencing these concepts. Therefore, this study will formulate a new theory that elucidates the determinants of the PU-process and PU-outcome concepts. Consequently, the grounded theory approach is highly suitable for this study.

The coding process was conducted following the guidelines of Holton and Walsh [44]. Which involves a systematic and iterative analysis of interview responses to identify patterns, relationships, and themes.

The coding process consisted of three stages: open coding, axial coding, and selective coding. During open coding, we divided interview transcripts into individual sentences, identified distinct concepts and ideas, and assigned codes to individual sentences. The axial coding stage aimed to group qualitative data into categories and subcategories based on similarities and differences, while selective coding involved refining and consolidating the categories and subcategories generated in the axial coding stage. The core categories that explained the data were then identified.

As the coding process is iterative, we continually reviewed and adjusted the codes and categories to ensure that they accurately reflected the data. Meetings were held to resolve discrepancies in opinions between coders, and more than five rounds of coding and recoding were conducted due to the large volume of qualitative data and complexity of open coding. Although the accuracy of the coding was attempted to be calculated using Cohen's Kappa value, it was abandoned due to the excessively complex process.

## 4 Findings

### 4.1 RQ 1: For pre-service ESL teachers, what are the connotations of seewo IWB's perceived usefulness in enhancing learning process (PU-process)?

In analyzing the qualitative data, we divided it into individual sentences, with each conveying a single piece of information. The 16 focus groups provided 59 pieces of information regarding the perceived usefulness of Seewo IWBs in enhancing the learning and teaching process (PU-process).

Specifically, participants believed that the Seewo IWB is advantageous for students as it enhances teacher-student interactions, captures their attention, promotes learning engagement, and alleviates learning anxiety. Furthermore, the Seewo interactive whiteboard can also be beneficial for teachers, as it assists in accessing learning materials, diversifying knowledge presentation, and emphasizing key information.

**Enhancing teacher-student interactions.** The significant majority of focus groups, 13 out of 16 to be specific, indicated that IWB effectively improve teacher-student interactions. Participants believed that these interactions were primarily facilitated through the self-contained voting system in Seewo Pinco. A voting system, also known as a student response system (SRS), classroom response system (CRS), or clickers, is a combination of software and hardware that allows students to submit multiple-choice answers using a transmitter. This transmitter sends the response through radio frequency, which is then captured by a computer via a USB receiver and displayed on a screen.

G6 noted that in traditional large-class lectures, teachers often use demonstrative tools, such as Microsoft PowerPoint, to present pre-set teaching content. In this approach, “teachers rarely adjust teaching content according to learners' needs” (G6). Utilizing a voting system enables teachers to accurately gauge students' understanding of the material and identify areas that require further emphasis (G5, G6, G8, G14). As a result, teachers can promptly provide necessary guidance. The ability to understand and respond to students in real-time is a hallmark of effective cognition-level teacher-student interactions (G2, G8, G12, G14). Additionally, G16 mentioned that the use of IWBs offers more opportunities for students to practice oral English with teachers, though they did not elaborate on specific methods.

**Capturing learners' attention.** The data revealed that Seewo IWBs play a significant role in capturing students' attention. G16 suggested that Seewo IWBs easily attract attention because they present a combination of pictures, text, sounds, and images. Three other groups noted that the technology is effective in showcasing knowledge through digital games, catering to students’ learning interests (G1, G8, G9).

**Enhancing learning engagement.** Seewo IWBs were also reported useful in enhancing learning engagement. Participants expressed a strong interest in the game-like activities provided in Seewo EasiNote. A member of G5 described one such activity as follows:

“ … the screen is divided into two left-and-right sections, each displaying a cartoon character. When the game starts, the characters begin to run. Two students can stand in front of the board and interact with it using their fingers, selecting the correct answer to questions. If they are correct, the character continues running; if incorrect, the character falls and cries. Points are also awarded or deducted accordingly. Throughout the process, the software provides background music to create an upbeat atmosphere."

Some participants attempted to analyze the reasons behind this increased engagement. G7 suggested that Seewo IWBs are engaging due to the intentional incorporation of eye-catching “wow factors” (G7) and the refreshing game-like design (G8, G12). Combined with sound effects, a typical game element that enhances engagement, Seewo IWBs become a valuable tool that “immediately increases learning interests” (G5).

It is important to note that “attracting attention” and “enhancing learning engagement” are two overlapping concepts in the current study. During interviews, participants used these terms interchangeably. The coding of these two concepts was somewhat intertwined, although this had minimal impact on the overall findings.

**Reducing students' learning anxiety.** Three groups highlighted the benefit of decreasing learning anxiety when using Seewo IWBs (G6, G7 and G12). G7 stressed that “the brainstorm function in Seewo Pinco encourages students to voluntarily participate in classroom activities, freeing them from the anxiety of ‘being caught unprepared”. G6 expressed appreciation for the anonymity mechanism provided by the voting system, stating that it “protects students' self-esteem” and “reduces anxiety”. G7 added that when using the voting system, “my peers won't know whether I have selected the wrong answer”, which also helps alleviate stress.

**Accessing more teaching materials**. Four groups mentioned that the Seewo IWBs offer a wealth of teaching resources, as users can easily access materials on the Internet (G4, G7, G8, G10). Participants noted that while multimedia systems are common in Chinese secondary schools, allowing teachers to access computers, in most cases, “teachers still need to connect to the Internet manually” (G8). This process “can be troublesome and time-consuming” (G8). G8 explained that if a classroom is equipped with an interactive whiteboard, accessing the Internet “would definitely be better."

**Diversifying information presentation.** Five groups suggested that the Seewo IWB facilitates the diversification of knowledge presentation (G5, G8, G9, G14, and G16). Specific knowledge points can be easily presented using mind maps (G5), pictures (G5), animations (G9), videos (G5), multimedia courseware (G14), matching games (G8, G9), and more. In summary, “there are numerous ways to present the teaching content” (G16).

**Highlighting key information.** Emphasizing key points and challenging concepts is a crucial aspect of teaching and serves as a yardstick to measure teaching effectiveness. In traditional blackboard-only classrooms, highlighting is typically achieved by changing chalk color or underlining the target information. In the PowerPoint-based multimedia era, highlighting is mainly accomplished by changing text colors or adding eye-catching animations. The interactive nature of IWBs provides more options: teachers can easily achieve a “zoom-in” effect by placing two fingers on the electronic whiteboard and moving them in opposite directions (G2), or by using the magnifying glass offered in the Seewo EasiNote toolkit (G8). Additionally, the interactive nature of IWBs enables users to directly leave marks on the board, making real-time annotation for highlighting incredibly convenient (G4, G8).

### 4.2 RQ 2: For pre-service ESL teachers, what are the connotations of seewo IWB's perceived usefulness in enhancing learning outcomes (PU-outcome)?

As highlighted in the Introduction section, PU-outcome refers to the perceived usefulness of Seewo IWBs in improving teaching and learning outcomes. Participants in the current focus group study reported 60 pieces of information regarding the PU-outcome of the IWB. The Seewo IWB facilitates knowledge understanding, ensures pronunciation correctness, cultivates students’ awareness of time management, save class delivery and class preparation time, promotes innovative teaching, and facilitates classroom management.

**Facilitating knowledge understanding.** Half of the focus groups indicated that Seewo IWB can facilitate knowledge understanding. Firstly, as a device connected to a computer, IWB enables users to use videos and pictures to better present knowledge points, which consequently simplifies knowledge comprehension (G4); secondly, as an interactive screen, IWB enables students to draw on the screen for hands-on learning experiences (G2); thirdly, the toolkit in Seewo EasiNote provides tailored tools for subject teaching, such as the periodic table of elements for Chemistry learning, the four-lined worksheet for English handwriting, and the English-Chinese dictionary for vocabulary acquisition (G1, G4, G6). The “variety of intelligent tools” are customized for easier subject teaching, which, in turn, “helps students understand the learning concepts better” (G4). Overall, the Seewo IWB provides multiple means to simplify or diversify knowledge presentation, as well as opportunities for hands-on experiences.

**Ensuring pronunciation correctness.** “Actually, many ESL teachers cannot pronounce English words properly.” Commented a participant in the fourth focus group. As a result, participants expressed appreciation for the English-Chinese dictionary embedded in Seewo EasiNote, which helps create flashcards, each containing the word, corresponding phonetic symbols, and a horn-like pressable electronic icon for standard English pronunciation (G4, G7, G9, G10). This is a typical, simple-yet-useful function for ESL learning.

**Cultivating students’ awareness of time management.** Two groups mentioned that they appreciate the “countdown clock” in Seewo EasiNote, stating that it reminds students that time is limited and that they should strategically allocate their time to different tasks (G16). This ultimately improves "students' awareness of time management” (G13).

**Saving class delivery and class preparation time**. Participants reported that IWB helps save both class delivery time and class preparation time. Two groups, G1 and G4, mentioned that knowledge presentation via an IWB is more efficient than blackboard writing. Meanwhile, “using fingers, teachers can quickly drag and enlarge knowledge points” (G7), as well as erase them (G3). Five groups appreciated Seewo IWB for its ability to save class preparation time. Using Seewo EasiNote, ESL teachers can prepare educational materials, share them, or download others' and make modifications (G4, G10, G11, G12).

**Promoting innovative teaching.** Notably, ESL pre-service teachers mentioned an important idea: using IWBs encourages teachers to adopt innovative teaching methodologies. According to the qualitative responses, IWBs are useful for student-centered learning (G9), cooperative learning (G10, G12), project-based learning (G7), layered teaching (G3), experiential learning (G8), and more. When standing in an IWB-equipped classroom, teachers naturally feel that they should employ innovative teaching methodologies or technologies. Even if teachers may not always succeed in doing so, the boosted motivation is valuable.

**Facilitate classroom management.** Facilitate Classroom Management: Surprisingly, Seewo IWB as a teaching tool is also recognized as useful in classroom management. G14 said that in Seewo EasiNote there is a “noise maker” to remind students to keep quiet. Additionally, the video-recording function of the Seewo system can be used to remind students that “the teacher is watching,” so students can be more disciplined while taking classes (G14). G14 commented that it is “especially useful for younger children with poor self-discipline.” Using a camera to record students' behaviors can be controversial, as it might violate students' privacy; however, maintaining order in the classroom is also an aspect that makes IWBs perceived as useful.

### 4.3 RQ 3: For pre-service ESL teachers, what are connotations of seewo IWB's perceived non-usefulness?

Data analysis also revealed a theme that was not covered by the PU-outcome and PU-process concepts, which was named as “perceived non-usefulness”. Perceived non-usefulness also contains sub-themes, as detailed in Table 1.

Participants reported that IWB can be too entertaining to be educational, or even harmful to serious learning. Three groups believed that the Seewo IWB does not balance its entertainment nature and educational functions (G10, G11, G12). Namely, the integration of “wow factors” with educational content is poor. Four groups reported that the Seewo IWB can be distracting, as students may become more interested in the fancy factors rather than the “exhausting” educational content (G1, G10, G11, G12). G2 added that students tend to be immersed in the fun activities, and teachers need extra efforts to “drag them back to knowledge learning.” Other participants (n = 2) believed that the Seewo IWB itself is defective in integrating fun elements with educational content (G9, G13). G13 explained that they feel vulnerable when using the Seewo IWB to teach complicated or abstract content. Instead, they tend to use it to present knowledge points only, just like using Microsoft PowerPoint. G9 held similar viewpoints, commenting that the game-like interactive activities are so “superficial” that they can only be suitable for younger students, such as pupils.

Participants also expressed doubts about the learning quality when using IWB. G6 criticized that the use of IWB “can be a barrier for in-depth learning” since students tend to be overexcited for “ranking high on the speed-answer leaderboard, so the thinking process is neglected".

Participants also doubted the efficiency of using IWB for classroom-based teaching. G12 noted that to maintain engagement, teachers are expected to design multiple engaging activities, which is “effort-demanding.” They added that if teachers use the time and effort to employ other teaching approaches or use other educational technologies, the effectiveness could be better.

Although interactive whiteboards are expected to provide high-quality interactions, the actual interactions in the study were not in-depth or meaningful. G12 noted that the game-like activities provided by Seewo EasiNote only involve a limited number of learners, leaving the majority of students watching what happens. The low participation rate can be addressed by using the voting system provided in Seewo Pinco, which was highly appreciated by participants. However, G9 criticized using the voting system as it can be like forcing students to participate in traditional ‘pick-and-answer’ activities, particularly for shy or inexperienced students. This can render interactive teaching meaningless (G13).

Participants also questioned the suitability of Seewo IWB for different types of students. Besides different grades, students who are shy, quiet, or introverted may find it difficult to participate in group activities (G9). Additionally, G13 believed that interactive activities can be upsetting for underachievers, although the reasons were not elaborated.

Generally speaking, during the investigation into pre-service ESL teachers' perceptions of the usefulness of Seewo interactive whiteboard, a prominent theme of “perceived non-usefulness” emerged from the focus group discussions. The participating pre-service ESL teachers expressed negative views regarding the Seewo IWB's effectiveness in facilitating English language learning:

G13: “It has a negative impact on learning.”

G11: “We hold negative viewpoints of the usefulness of Seewo IWB.”

G16: “Some of the Seewo IWB functions cannot facilitate English learning.”

G16: “What was improved is ***interest*** rather than ***learning interest***. The two concepts are different.”

However, notably:

“Although lots of problems need to be addressed, the Seewo IWBs are significant in boosting learning engagement. This is thought-provoking”. (G16)

Findings of the current study are summarized in Table 2 below.

**Table 2:** Summary of ESL teachers’ qualitative responses.

Theme	Sub-theme
PU-process	1) Improve teacher-student interactions 2) Attract attention 3) Enhance learning engagement 4) Reduce learning anxiety 5) Enable teachers to get access to more teaching materials 6) Diversify knowledge presentation 7) Highlight key information
PU-outcome	1) Facilitate knowledge understanding 2) Ensure pronunciation correctness 3) Cultivate students' time management awareness 4) Save class-delivery time 5) Save class-preparation time 6) Promote innovative teaching 7) Facilitate classroom management
Perceived non-usefulness	1) Distract students 2) Hinder students from in-depth thinking 3) Be time-consuming or effort-demanding 4) Cause superficial or meaningless interactions 5) Be suitable for a limited number of students

## 5 Discussions

### 5.1 Whether the perceived usefulness outweighs the perceived non-usefulness?

Previous studies reported multiple benefits of interactive whiteboard, such as increasing interactions [3,5,31], increasing learning engagement [1,25], enhancing learning efficiency [30,31], getting access to more resources [6], promoting learner-centered learning [25], involving tactile or kinesthetic learners [26,30], saving time for class preparation [32].

However, an obvious theme emerged in the current study: perceived non-usefulness. The participants commented that Seewo IWB “cannot facilitate English learning” (G16) or “negatively impacts on learning” (G13). To be specific, participants reported that the students can be distracted by the “wow factors”, so teachers need extra effort to bring students back to learning status. Most importantly, students’ curiosity and interest can quickly drop to the normal level once they got familiar with the stimuli, but IWB fails to provide new stimuli [33].

Teachers also have a tendency of using interactive whiteboard as a conventional screen [30,33] or a “game” [34], so IWB's potential could be limited. As criticized by Luo [35], superficial use of technologies brings distractive or even opposite effectiveness results. Consequently, an interactive whiteboard can be perceived significantly useful if properly utilized, while being perceived un-useful if being used as a screen or a game.

In conclusion, firstly, interactive whiteboards (IWBs) possess the potential to significantly enhance short-term engagement, although their capability to ensure a lasting improvement in learning quality remains uncertain. It is evident that, when using IWBs, educators need to focus on the purpose behind their usage: whether it is solely to enhance students' test-taking abilities or to also elevate their interest and learning experience. If the latter holds significant importance, then using IWBs in ESL teaching is worthwhile.

Secondly, IWBs can provide a range of extrinsic motivations through points, badges, and sound effects, yet these might lead to distraction and a decrease in intrinsic motivation. Hence, teachers need to foster intrinsic motivation. There are various ways to provide intrinsic motivation, such as offering learning materials highly relevant to students (relatedness), preparing educational activities of appropriate or slightly elevated difficulty matching students' levels (competence), addressing topics of student interest (motivation to know), offering timely feedback and encouragement (motivation to be recognized), and enabling students to act independently and feel that their efforts hold significance (motivation to accomplish).

Thirdly, training is pivotal for the effective utilization of IWBs in ESL instruction. When educators perceive IWBs as beneficial to ESL teaching and have acquired the necessary operational skills, they are more inclined to utilize the advanced functionalities, thereby more effectively unleashing the potential of IWBs.

### 5.2 Why perceived non-usefulness is an obvious theme? What could be the contributing factors?

Research results indicate that users' perceived usefulness of IWBs is inconsistent, with some even believing that IWBs can have negative effects.

Analysis has revealed that one fundamental reason for this phenomenon is the neglect of the cost-effectiveness concept. Cost-effectiveness, defined as the ratio of effect to cost, takes into account both desired outcomes and monetary inputs [45]. As G12 stated, to utilize IWBs to deliver a genuinely engaging and effective class, teachers need to design multiple and diversified activities, which can be time-consuming and effort-demanding. The issue is that, “if teachers use the time and effort to employ other teaching approaches or use other educational technologies, the effectiveness could be better”. This is consistent with previous studies, such as Zhou, Li and Wijaya [39], who reported that electronic interactive whiteboards may not be as labor-saving, resulting in no significant effect on the final acceptance intention. It is evident that people recognize the motivational function of IWBs, but cost-effectiveness concerns overshadow it.

The concept of cost-effectiveness is highlighted in the study of Luo, Brown and O'Steen [24], which investigates ESL teachers' acceptance of gamified educational tools. The study participants acknowledged the benefits of this innovative technology, but the concern lies in the significant investments required to fully realize its potential in terms of time, money, and human resources. Thus, while the IWB tool itself has a significant impact on student learning, its advantages are not as apparent when compared to other similarly priced educational tools.

It is worth noting that there is a certain research bias in this step: the interviewees were ESL student teachers, not school decision-makers who need to decide whether to pay for IWBs. As far as we know, in China at least, there is a significant amount of funding allocated for promoting the use of educational technologies in schools, and currently, a large number of schools have installed electronic interactive whiteboards. For these schools, the key issue is not whether to purchase IWBs, but how to make the most of them. Therefore, the issue of cost-effectiveness may be a hypothetical problem raised by ESL student teachers. To address any resulting technology resistance, the solution is simple: realizing that the investment has already been made, using IWBs more often will result in more benefits.

Cost-effectiveness is a concern not only when comparing IWB with other educational technologies, but also during its usage. The study was conducted in a smart classroom equipped with multiple Seewo IWB-related or supporting products, with a total value exceeding ten thousand US dollars. However, despite the availability of advanced technology, teachers still tend to use IWB as a regular blackboard, with the only difference being the use of fingers to leave marks on the IWB-based smart board instead of chalk or marker pens on a traditional blackboard.

This behavior is related to teachers' workflow, beliefs, and personal innovativeness. According to Rogers (2003), if a new product does not align with an employee's work habits or beliefs, its acceptance rate will be low. Furthermore, individuals with low personal innovativeness may struggle to accept new products or may only adopt them after they become popular among others. Age is also a factor that influences personal innovativeness.

These findings suggest that providing relevant training on new technologies to teachers as early as possible, preferably during pre-service training, can increase the likelihood of adoption. Pre-service teachers are still forming their workflows and developing teaching beliefs, while their personal innovativeness tends to be higher due to their younger age. Thus, early exposure to new teaching tools can help teachers incorporate them into their practice effectively.

One way to promote technology acceptance is through increased training, which is the approach taken by most schools and companies to support teachers. However, previous literature has generally emphasized the need for teacher training without specifying what type of training is required [1,2,34,46]. In this study, participants expressed concerns about the applicability of electronic whiteboards. They found it challenging to use the IWB to enhance learning experience or learning outcome when teaching abstract knowledge, in-depth discussions, or older students. They also noted that even if they were familiar with the IWB's various functions, integrating the new technology with pedagogical content was a significant challenge, and the available tutorials were insufficient.

These findings indicate that IWB training for teachers should not only focus on the technology's features but also provide more direct examples that incorporate pedagogical content. These examples should also consider different types of knowledge, teaching depths, and student types.

Teacher instructors should impart crucial awareness to pre-service teachers: not to get carried away by the wow factors of new technology, to comprehend that different functions have varying applicability, and to use certain functions selectively. The main focus should be on their teaching objectives and design, using the IWB as an aid to teaching rather than allowing it to take control.

### 5.3 Whether the IWB concept be limited to a hardware-related one?

The results also indicate that some evaluations of the usefulness of IWB are based on software, rather than hardware. By definition, an interactive whiteboard should be a large touch-sensitive board that is connected to a computer and a projector [5,6,20], namely a piece of electronic hardware. During data collection, participants presented a tendency of enlarging the IWB concept: even though the researchers stressed that Seewo Pinco is a piece of supplementary software, participants still tend to name Seewo Pinco as “Seewo interactive whiteboard”. The consequence is obvious: the interactivity-related responses were mainly about the voting system provided by the software Seewo Pinco, rather than the interactions between students and the touch-sensitive screen.

During the discussion, participants noted that Seewo EasiNote offers a timer function, which can help cultivate time management skills among students. Additionally, the software's camera feature enables real-time video capture of the entire classroom, allowing students to review their actions and ultimately contributing to better classroom discipline. These unique perspectives shed new light on the benefits of the software.

The results suggest that IWB can expand beyond the hardware concept by incorporating additional supplementary software. Classroom resposne systems and open educational resources can be added to the supplementary software [23,27,33].

### 5.4 Whether engagement can be sustained in learning activities using IWB?

The question of whether interactive whiteboards (IWBs) can sustain engagement in learning activities is a topic of ongoing discussion [35]. Many publications have criticized this issue, as seen in titles like *Interactive whiteboards: Interactive or just whiteboards?* [20], *Interactive whiteboards: Real beauty or just ‘‘lipstick”?* [3], *Interactive whiteboard for primary schools in Mauritius: An effective tool or just another trend?* [47] and *Interactive whiteboards: boon or bandwagon? A critical review of the literature* [6]. In the current study, participants reported the same concern: “very soon the students get bored” (G10).

Interestingly, previous studies even suggested that the use of IWBs can actually lead to decreased student engagement, as teachers who are enthusiastic about IWBs tend to monopolize the board, leaving less time for student-screen interactions [20,33].

Luo [35] has identified various factors that can influence the effectiveness of educational technology, such as user resistance, inappropriate use, poor integration with pedagogical content, ignorance of the novelty effect, and short experimentation timeframes. To address the sustainability of engagement with IWBs, it is essential to consider whether ESL teachers have truly accepted the technology, whether it is being used appropriately, whether it is well-integrated with pedagogical content, and whether the novelty effect has been adequately addressed through long-term use. Providing incentives can also be an effective way to motivate students over the long term.

### 5.5 What others can be considered in IWB studies?

There are several additional factors that should be considered in IWB studies. For example, the use of IWBs can be challenging for anxious students, as noted by Kühl and Wohninsland [48], and may not be suitable for shy or introverted students, as reported by participants in this study. Individual differences should always be taken into account [29].

Another overlooked research gap is the identification of change agents that can influence the acceptance of IWBs. Previous studies have identified several factors that hinder users' acceptance of educational technologies, such as high cost, lack of technical support, insufficient teacher knowledge, inadequate teacher training, low relatedness to conventional teaching, insufficient pedagogical values, incompatible pedagogical beliefs, overlooked interactivity, and low ease-of-use [1,16,20,26,30,31,33,34,37,46,49]. However, it is essential to identify who can drive change in the context of IWB acceptance. Is it the IWB companies, school principals, educationalists, or in-service teachers? If the change agents are not specified, the acceptance of IWBs may be hindered.

In summary, the IWB concept should not be limited to a hardware-related one. Instead, designers can add more supplementary software to the IWB hardware. Whether IWB can provide long-term engagement is an issue, as well as the benefits of getting access to massive online resources. Besides, it is hard to say whether IWBs’ perceived usefulness outweighs the perceived non-usefulness, so further explorations are needed.

## 6 Conclusion

### 6.1 Summary and significance

This study aims to explore how pre-service ESL teachers understand the perceived usefulness of the Seewo interactive whiteboard. The authors recruited 80 ESL teachers, divided them into 16 groups, conducted a focus group interview to collect data, and analyzed the data using grounded theory techniques.

Three research questions were answered: for pre-service ESL teachers, what are the connotations of Seewo IWB's perceived usefulness in improving learning process (PU-process), what are the connotations of Seewo IWB's perceived usefulness in enhancing learning outcomes (PU-outcome), and what are connotations of Seewo IWB's perceived non-usefulness.

This study has obtained valuable findings. Firstly, the concept of perceived usefulness should be divided into two parts: perceived usefulness in improving the learning process (PU-process) and perceived usefulness in enhancing learning outcomes (PU-outcome). IWBs were reported to be significant in PU-process, but cannot guarantee PU-outcome. In order to ensure PU-process, teachers should not focus too much on the entertainment provided by IWBs, in order to avoid distracting students, nor should they treat IWBs as ordinary information presentation tools. That is, teachers need to learn how to use this new educational tool better in ESL classrooms, otherwise the usefulness of IWBs will be greatly restricted.

### 6.2 Limitation

One major limitation of this study is that some participants came in with preconceptions. Specifically, some assumed that the IWB was already available, and therefore did not express any financial concerns. On the other hand, some participants denied the usefulness of IWBs because they assumed that their school did not have the funds to purchase them. In future studies, it is important to address this issue by taking into account the diversity of participants' backgrounds.

### 6.3 Recommendations for future research

There are several areas for improvement in this situation. Firstly, recognizing the importance of cost-effectiveness and understanding that IWBs only provide benefits if used frequently. Secondly, providing IWB training at the beginning of teacher training to ensure that educators can use the technology effectively. Additionally, providing practical teaching operation cases that cater to different knowledge types, depths of teaching forms, and student types is crucial. It is equally important for teacher instructors to encourage pre-service teachers to focus on their own teaching designs and use IWBs as a helpful tool, rather than a restrictive one.

The IWB industry can develop more software to create interactive learning experiences, while academic research should consider the sustainability of engagement and whether IWBs are being used properly and integrated with pedagogical content.

Future studies are also recommended to involve software to better support interactive teaching. More attention should be paid to the assessment of IWB's effectiveness [48], especially the negative ones in the long run. Future IWB studies can also consider cost-effectiveness and learner styles.

## Ethics considerations

All procedures performed in studies involving human participants were in accordance with ethical standards. Informed consent was obtained from all individual participants included in the study.

## Funding information

This study is funded by 10.13039/100010338Chongqing Normal University. The project ID is: 22XWB002.

## Author contribution statement

Zhanni Luo: Conceived and designed the experiments; Analyzed and interpreted the data; Wrote the paper.Xiaoyong Tan: Performed the experiments. Mu He, Xianyou Wu: Contributed reagents, materials, analysis tools or data.

## Data availability statement

Data will be made available on request.

## Additional information

No additional information is available for this paper.

## Declaration of competing interest

The authors declare that they have no known competing financial interests or personal relationships that could have appeared to influence the work reported in this paper.

## Abbreviations

ESL: English-as-the-second-language
IWB: interactive whiteboard
PU: perceived usefulness
PEoU: perceived ease of use
TAM: Technology Acceptance Model
